# Expression Profile of Long Noncoding RNAs and Circular RNAs in Mouse C3H10T1/2 Mesenchymal Stem Cells Undergoing Myogenic and Cardiomyogenic Differentiation

**DOI:** 10.1155/2021/8882264

**Published:** 2021-04-30

**Authors:** Mingshan Li, Zijie Pei, Hongtao Zhang, Jing Qu

**Affiliations:** ^1^Department of Cell Biology, Medical College of Soochow University, Soochow University, Ren Ai Road 199, Suzhou Industrial Park, Suzhou 215123, China; ^2^Department of Orthopedics, The First Affiliated Hospital of Soochow University, Suzhou, Jiangsu, China

## Abstract

**Background:**

Currently, a heterogeneous category of noncoding RNAs (ncRNA) that directly regulate the expression or function of protein-coding genes is shown to have an effect on the fate decision of stem cells. However, the detailed regulatory roles of ncRNAs in myogenic and cardiomyogenic differentiation of mouse C3H10T1/2 mesenchymal stem cells (MSCs) are far from clear.

**Methods:**

In this study, 5-azacytidine- (5-AZA-) treated C3H10T1/2 cells were differentiated into myocyte-like and cardiomyocyte-like cells. Next, ncRNA associated with myogenic and cardiomyogenic differentiation was identified using high-throughput RNA sequencing (RNA-seq) data. Bioinformatics analysis was conducted to identify the differentially expressed ncRNAs and the related signaling pathways.

**Results:**

Myotube-like structure was formed after 5-AZA treatment of C3H10T1/2 cells. In addition, myogenic and cardiomyogenic differentiation-related genes like *GATA4*, *cTnt*, *MyoD*, and *Desmin* were upregulated significantly after the 5-AZA treatment. Totally, 1538 differentially expressed lncRNAs and 3398 differentially expressed mRNAs were identified, including 1175 upregulated and 363 downregulated lncRNAs and 2429 upregulated and 969 downregulated mRNAs. In addition, 46 differentially expressed circRNAs were identified, including 25 upregulated and 21 downregulated circRNAs. Moreover, the differentially expressed mRNAs were enriched into 5 significant pathways, including those for focal adhesion, ECM-receptor interaction, PI3K-AKT signaling pathway, PPAR signaling pathway, and Tyrosine metabolism.

**Conclusions:**

A systematic view of the expression of ncRNAs in myogenic and cardiomyogenic differentiation of MSCs was provided in the study.

## 1. Introduction

Mesenchymal stem cells (MSCs) are a kind of pluripotent stem cells, which are now frequently used in regenerative medicine research, capable of self-renewal and differentiation into other cell lineages, such as myoblasts [1], adipocytes, chondrocytes, osteoblasts [2], and possess several specific features, which make them important candidates for future regenerative therapies. Accumulating studies have shown that transplantation of MSCs with myogenic potential could regenerate skeletal muscle where they engrafted in mouse models of acute and chronic muscle association injury [3]. In addition, in both ischemic and nonischemic cardiomyopathy, MSCs have the potential to improve cardiac function and reduce infarct size [4, 5]. Many studies have used MSCs for the identification of involved mechanisms, drug screening [6], and gene correction studies. The rationale of stem cell transplantation is to repair the damaged tissue by implanting cardiomyogenic cells or genes, with the expectation that the transplanted cells will contribute to generate new myocardial tissue. However, many challenges must be addressed for stem cell-based therapy, including increasing differentiation efficiency, improving cell retention and survival, and reducing immune rejection.

Many agents such as cytokines, growth factors, and small molecules have so far been applied to induce stem cells into myocytes [7]. It is of great significance to find a method to achieve a high differentiation rate and low cytotoxicity for stem cell therapy. 5-Azacytidine (5-AZA), a demethylation pharmaceutical, has the ability to induce MSCs into cardiomyocytes-like and myocytes-like cells [8], while the detailed mechanisms are not cleared yet. It has been reported that the differentiation of MSCs is regulated by a series of signaling cascades. Studies have shown that epigenetic modifications play important roles in MSC differentiation into cardiomyocytes [9]. 5-AZA is thought to be one of the significant inducers, activating some dormant genes by demethylation to induce expression of heart-specific markers, which is consistent with a myocyte lineage. However, the detailed mechanism underlying this process is unclear.

Myogenic and specification and differentiation of the heart are complex biological processes determined by the activities of gene regulatory networks. At the molecular level, emerging studies have confirmed the role of long noncoding RNAs (lncRNAs) in controlling major biological processes that affect stem cell differentiation and development [10, 11]. For instance, it was shown that lncRNA *Fendrr* and Braveheart (*Bvht*) is essential for proper fate control of lateral mesoderm derivatives [12]. lncRNA *Fendrr* play regulatory roles binds both TrxG/MLL complexes and the polycomb repressive complex 2 (PRC2), while *Bvht* interacts with SUZ12 during cardiomyocyte differentiation in the mouse. In another study, lncRNA *CARMEN* was shown to interact with SUZ12 and EZH2, two components of PRC2, and act as upstream of the mesoderm-specifying gene regulatory network [13]. lncRNAs can act as a signal, guide, or scaffold for chromatin and regulate epigenetic gene expression by interacting with chromatin modification or remodeling factors [14]. Circular RNAs (circRNAs) are one class of noncoding RNAs widely found in animal cells. circRNAs could mediate the activity of microRNAs (miRNAs) through binding and functioning. More and more evidence has shown that circRNAs are often expressed abnormally under different physiological and pathological conditions and may contribute to stem cell differentiation and development through miRNAs [15, 16].

To promote muscle regeneration, it is important to improve our understanding of signaling pathways and molecular regulatory circuits such as ncRNAs that control the differentiation of stem cells to the muscle and cardiac lineage. The C3H10T1/2 cell line isolated from C3H mouse embryos has several characteristics, which were similar to MSCs. Previous studies have shown that a low concentration of DNA methylation inhibitor 5-AZA can convert C3H10T1/2 cells into skeletal muscles, adipocytes, and chondrocytes [17]. Due to the availability of C3H10T1/2 cells in vitro, the myoblast differentiation process is a useful system for studying the biological functions of ncRNAs. In this study, the expression profiles of ncRNAs during myogenic and cardiomyogenic differentiation of MSCs were identified and characterized to take the advantage of high-throughput RNA-sequencing (RNA-Seq). Our findings may potentially lead to the development of more effective therapeutic methods to muscle-related diseases such as Duchenne muscular dystrophy and heart diseases.

## 2. Materials and Methods

### 2.1. Differentiation of C3H10T1/2

**Figure 1:**
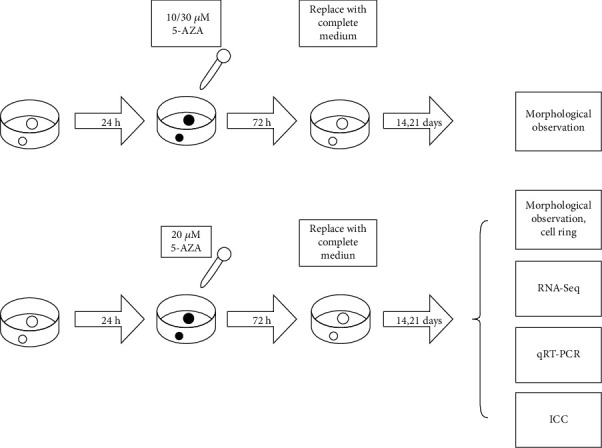
Reagent concentration and induction time point.

C3H10T1/2 mouse multipotent mesenchymal progenitor cells were obtained from the Cell Bank of China Science Academy (Shanghai, China). Briefly, C3H10T1/2 was cultured in Dulbecco's Modified Eagle Medium (DMEM) supplemented with 10% fetal bovine serum (FBS), 2 mM L-glutamine, 100 U/ml penicillin, and 100 *μ*g of streptomycin (1% Pen/Strep) at 37°C in 5% CO_2_. Cells were seeded in six-well plates at a density of 5,000 cells/ml 24 hours prior to inducing myogenic and cardiomyogenic differentiation. Then, the cells were incubated with 5-AZA (Sigma-Aldrich) at 10, 20, and 30 *μ*mol/L, respectively, and incubated for 24, 48, and 72 hours. Then, the cells were replaced with fresh growth media and incubated in a CO_2_ incubator for another 7-21 days. The medium was replaced in every 2-3 days, and morphological changes of the cells were observed at the same time. Reagent concentration and induction time point are shown in Figure 1.

### 2.2. RNA Preparation and Gene Expression Analysis

Total RNA was extracted from untreated and 5-AZA treated C3H10T1/2 cell samples (20 *μ*mol/L) by using TRIzol (Invitrogen Life Technologies, Carlsbad, CA, USA) according to the manufacturer's instructions. cDNA was prepared using a cDNA synthesis kit (Promega, Madison, WI, USA). Quantitative real-time PCR (qRT-PCR) analysis of mRNA expression was performed as described previously with normalization to GAPDH (glyceraldehyde-3-phosphate dehydrogenase) and carried out with SYBR supermix (Takara, Shiga, Japan). The primers used for gene amplification are shown in Table 1. On days 7, 14, and 21, cardiomyocyte-specific markers (*GATA-4*, *cTnt*, *α-MHC*, *Nkx2.5*, and *MEF2c*) and myogenic-specific markers (*MyoD*, *Myogenin*, and *Desmin*) of the untreated and 5-AZA treated C3H10T1/2 cells were identified by qPCR. The expression of each mRNA relative to GAPDH was calculated based on the threshold cycle (CT) as *r* = 2 − Δ(ΔCT).

### 2.3. RNA Sequencing

RNA was extracted from two untreated and two 20 *μ*mol/L 5-AZA treated C3H10T1/2 cell samples by using TRIzol. RNA from each sample (C3h10_1 and C3h10_2, untreated; C3h10_101 and C3h10_102, 20 *μ*M 5-AZA 5-AZA treated) was quantified and qualified by Agilent 2100 Bioanalyzer (Agilent Technologies, Palo Alto, CA, USA) and NanoDrop (Thermo Fisher Scientific Inc.). RNA integrity for each sample was assessed using standard denaturing agarose gel electrophoresis. 1 *μ*g total RNA of each sample with RIN value above 7 was used for library preparation. Libraries with different experimental groups were multiplexed and loaded on an Illumina HiSeq instrument according to the manufacturer's instructions (Illumina, San Diego, CA, USA). The sequences were processed and analyzed by GENEWIZ.

### 2.4. qRT-PCR Test RNA-Seq

We selected 8 lncRNAs and 3 circRNAs differentially expressed by RNA-seq (8 lncRNAs, 5 upregulated, 3 downregulated, 3 circRNAs, 2 upregulated, and 1 downregulated) for qRT-PCR verification. Total RNA was extracted from untreated and 5-AZA-treated C3H10T1/2 cell samples (20 *μ*mol/L) by using TRIzol (Invitrogen Life Technologies, Carlsbad, CA, USA) according to the manufacturer's instructions. cDNA was prepared by cDNA synthesis kit (RevertAid First Strand cDNA Synthesis Kit, Thermo Scientific, China). lncRNA and circRNA expressions were analyzed using Roche (Roche LightCycler96) qRT-PCR, as previously described, standardized with GAPDH (glyceraldehyde-3-phosphate dehydrogenase), and performed with SYBR Green I (RiboBio, Guangzhou, China). The expression of each mRNA relative to GAPDH was calculated based on the threshold cycle (CT) as *r* = 2 − Δ(ΔCT). The primers for the experimental design are shown in Table 2.

### 2.5. Immunocytochemistry

The day before the experiment, the disinfected small round glass slides were put into a 12-well plate and coated with gelatin. The gelatin was removed after the coating and dried. Cells were transferred to a 12-well plate at a suitable density and cultured overnight. The cells were fixed with 4% buffered paraformaldehyde (PFA, Solarbio, Beijing, China) for 1 h. After removing the fixed solution, the cells were washed with PBS. 0.1% Triton X-100 (BioFroxx, Germany) and were used to make the cell membrane permeable. The samples were sealed in PBS with 1% Bull Serum Album (BSA, Solarbio, Beijing, China) for 60 min, and Desmin Ab-AF5334, TNNT2 Ab-DF6261, MyoD1 Ab-AF7733, and GATA4 Ab-AF5245 were diluted into 1% BSA at 1 : 100 and treated overnight at 4°C. After removing the primary antibody, the cells were washed twice with PBS. The cells were incubated with the secondary antibodies (Alexa Fluor® 488 AffiniPure Goat Anti-Rabbit IgG (H + L), Yeasen, Shanghai, China) for 2 h and then incubated with Hoechst 33342 (C1025, Beyotime, China) for 5 min. Between each step, the cells were carefully washed with PBS 3 times. For quantification, a fluorescence microscope (Leica DMi8, Leica Microsystems Heidelberg GmbH, Germany) was used to capture at least 8 nonoverlapping images of each marker. Cells positive for secondary antibody staining were counted as a percentage of the total cell count. The Colocalization_Finder plug-in of ImageJ (National Institutes of Health, Bethesda, MD, USA) was used to analyze the positive rate of cells. Each experiment was repeated more than five times. Data are expressed as mean ± SEM deviation.

### 2.6. Bioinformatics Analysis

Data were treated with Trimmomatic (v0.30) to remove technical sequences and to obtain high quality clean data. DESeq Bioconductor package was used to perform differential expression analysis. The *P* value of genes was set to <0.05 and adjusted by Benjamini and Hochberg's approach for controlling the error discovery rate. Gene Ontology (GO) terms and KEGG (Kyoto Encyclopedia of Genes and Genomes) analyses were used to annotate a rich list of genes and the related signaling pathways. We also performed GSEA analysis on differential expression genes to further explore the pathway of the target gene enrichment. CPC (coding potential calculator) was used to do protein-coding potential prediction. lncRNA-mRNA coexpression network analysis was formed by software Cytoscape. The differentially expressed circRNAs were detected by using negative binomial distribution test based on DESeq software package. By analysing the circRNAs with genetic elements, the distribution of circRNAs in the genome was investigated. Bioinformatics analysis was carried out by Oebiotech.

## 3. Results

### 3.1. Morphology and Characterization of Mouse Mesenchymal Stem Cells and 5-AZA Treatment Increased Myodifferentiation and Cardiomyocyte Differentiation

**Figure 2:**
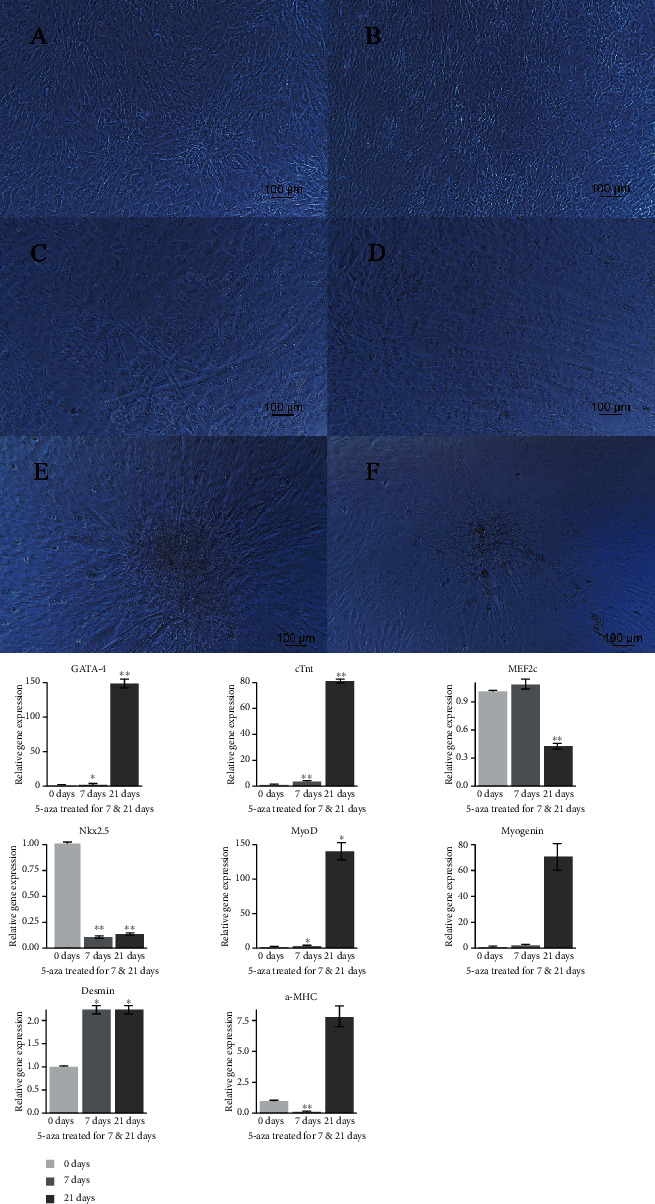
The morphology and characterization of mouse MSCs and the relative expression profile of cardiomyogenic and myogenic marker genes. Up: 5-azacytidine induces C3H10T1/2 cells into differentiated myocytes. (a) C3H10T1/2 cells. (b) C3H10T1/2 cells were treated with 20 *μ*M 5-azacytidine for 7 days, and cells were displayed in the same direction. (c and d) Myotube-like structure appeared after 5-azacytidine treating, then cells were changed into growth media and cultured for the next 21 days. (e and f) Differentiated myocytes were picked with cell ring and cultured for additional two weeks. Myocyte-like cells could be subcloned and had abilities to form myotube-like structure. Down: the relative expression profile of cardiomyogenic and myogenic marker genes: *GATA-4*, *cTnt*, *MEF2C*, *NKX-2.5*, *MyoD*, *Myogenin*, *Desmin*, and *α-MHC* were determined using real-time PCR at 0, 7, and 21 days after 5-azacytidine treatment (^∗^*P* < 0.05, ^∗∗^*P* < 0.01).

C3H10T1/2 mouse MSCs (Figure 2(a)) were cultured in six-well plates and treated with 5-AZA at 10, 20, and 30 *μ*M, incubated for 24 hours, respectively. The morphology of cells was changed dramatically after 7-8 days of culture. As shown in Figure 2(b), cube-like C3H10T1/2 cells were stretched into the spindle-like cells, displayed in the same direction, and arranged in parallel with higher refraction. Notably, myotube-like structure appeared gradually on these spindle-like cells following another 14 days culture. Basically, around 20-30% of the field-initiated myotube-like structure, a characteristic of mature myocytes following 21 days of 5-AZA treatment (Figures 2(c) and 2(d)).

To determine the efficiency of each 5-AZA concentration for myogenic induction, we compare the percentage of myotube-like structure and dead cell areas in each 5-AZA treatment group. Using 30 *μ*M 5-AZA treatment did not result in the highest induction efficiency, whereas this concentration results in a large amount of cell death. 10 or 20 *μ*M 5-AZA could induce morphology change and myotube-like structure effectively too. So, there is no direct correlation between the percentage of myotube-like structure and the concentration of 5-AZA. In addition, we notice that timeline is a very important thing for the 5-AZA induction process, cells always displayed morphology change after one week and myotube-like structure appeared after three to four week's culture. Alternatively, the cells were treated with 5-AZA for another 72 hours, which could improve the induction efficiency and this percentage approached 60-70% following 21 days of 5-AZA treatment.

To further evaluate the proliferation and growth characteristics of myotube-like cell structure, we picked myotube-like cells by using the tool of cell ring, subcloned, and cultured these cells for another two weeks. We found that these cells still have abilities to form myotube-like cells (Figures 2(e) and 2(f)), indicating that these cells have relatively stable differentiation status and limited proliferation abilities. However, spontaneous contractions are not appearing during all the culture processes.

qRT-PCR was conducted to detect the mRNAs expression level of specific myogenic and cardiomyogenic markers including *MyoD*, *Myogenin*, *Desmin*, *α-MHC*, *GATA-4*, *cTnt*, *Nkx2.5*, and *MEF2c*. The relative expression level of cardiomyogenic and myogenic marker genes was determined at 0, 7, and 21 days after 5-AZA treatment, respectively. As shown in Figure 2, the expression of *GATA-4* (^∗∗^*P* < 0.01), *cTnt* (^∗∗^*P* < 0.01), *MyoD* (^∗^*P* < 0.05), and *Desmin* (^∗^*P* < 0.05) were increased considerably in 5-AZA inducing groups, suggesting that 5-AZA plays important regulatory roles in MSCs differentiation into cardiomyocytes and myocytes. However, the expression level of *MEF2C*, *NKX-2.5*, *Myogenin*, and *α-MHC* was shown decreased or without changes (Figure 2 down).

**Figure 3:**
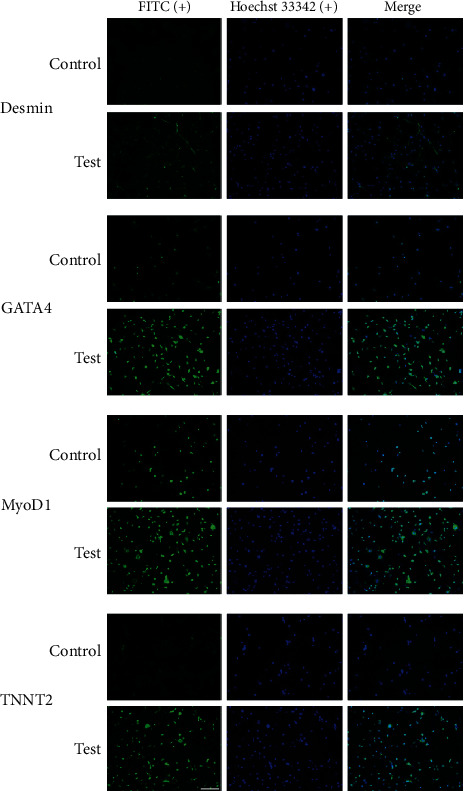
Markers related to myoblasts and cardiomyocyte differentiation were detected by ICC. 5-AZA induces a significant promotion of myogenicity and cardiomyocyte differentiation in MSCs. MSCs are fixed on petri dishes. Then, the sample was sealed and stained with antibodies against *Desmin*, *GATA4*, *MyoD1*, and *TNNT2*. The nuclei were then stained with Hoechst 33342. The positive proportion of staining was different in different groups (scale = 200 *μ*m).

To determine the effect of 5-AZA on C3H10T1/2 morphology and characterization differentiation, we performed MSC differentiation and immunocytochemistry experiments. After inducing myocyte differentiation and cardiomyocyte differentiation, the cells were stained with myogenic and cardio-myogenic differentiation-related markers *Desmin*, *GATA4*, *MyoD1*, and *TNNT2*, and the percentage of myocytes and cardiomyocytes in the differentiated cells was determined. The results showed that cardiogenic and myogenic specific markers *Desmin*, *GATA4*, *MyoD1*, and *TNNT2* were significantly upregulated after 5-AZA induction (Figure 3). Compared with the control group, the positive rate of *Desmin* was 64.2% ± 4.8% (^∗∗^*P* < 0.01), *GATA4* was 76.8% ± 5.6% (^∗∗^*P* < 0.01), *MyoD1* was 75.1% ± 4.3% (^∗^*P* < 0.05), and *TNNT2* was 76.6% ± 3.0% (^∗∗∗^*P* < 0.001) were shown upregulate expression significantly (data not shown). FITC (+)/Hoechst 33342 (+) quantitative analysis results were shown that the differentiation promoted the myogenic and cardiomyogenic differentiation greatly.

### 3.2. Identification of Differential Expression Profiles of mRNA-lncRNAs between Undifferentiated and Differentiated MSCs

**Figure 4:**
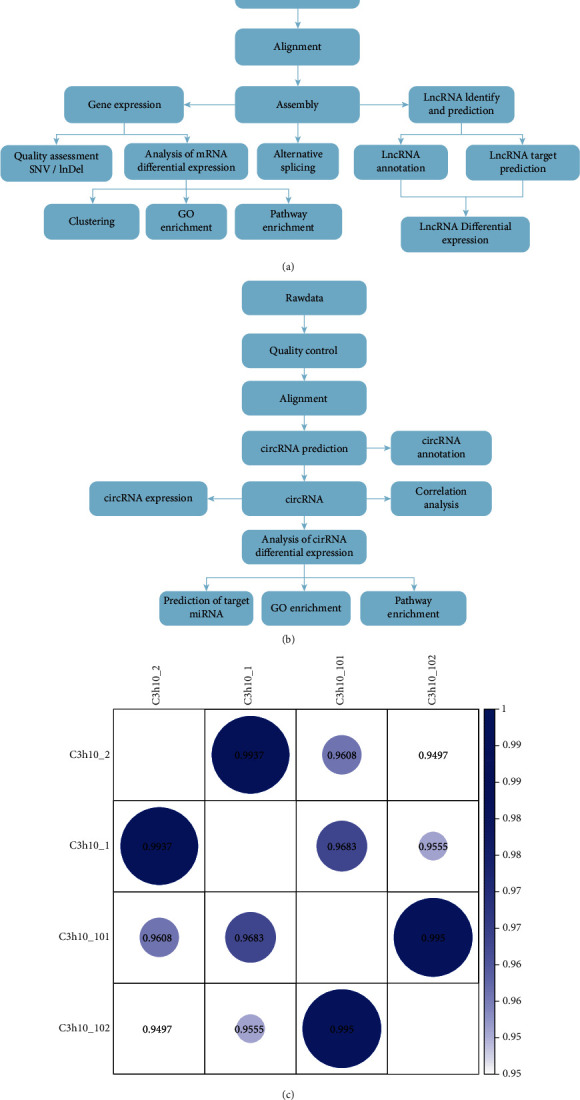
An overview of the genome-wide analysis of mRNA-lncRNA-circRNA study design and correlation coefficient analysis of two biological repeats for 5-AZA-treated and untreated cells. (a and b) An overview of the genome-wide analysis of mRNA-lncRNA-circRNA study design. (c) Correction between the two biological repeats for each sample (C3h10_1 VS C3h10_2: correlation index = 1; C3h10_101 VS C3h10_102: correlation index = 0.995).

Briefly, an overview of the genome-wide analysis of mRNA-lncRNA-circRNA study design is illustrated in Figures 4(a) and 4(b). To verify the correlation between the two biological repeats for each sample, we performed correlation coefficient analysis. As shown in Figure 4(c), there is a high correlation index 0.9937 between the two untreated groups: C3h10_1 and C3h10_2. For the two 20 *μ*M 5-AZA-treated group: C3h10_101 and C3h10_102, the correlation index is 0.995. These data indicate a positive correlation between the two biological repeats for 5-AZA treated and untreated cells, respectively.

**Figure 5:**
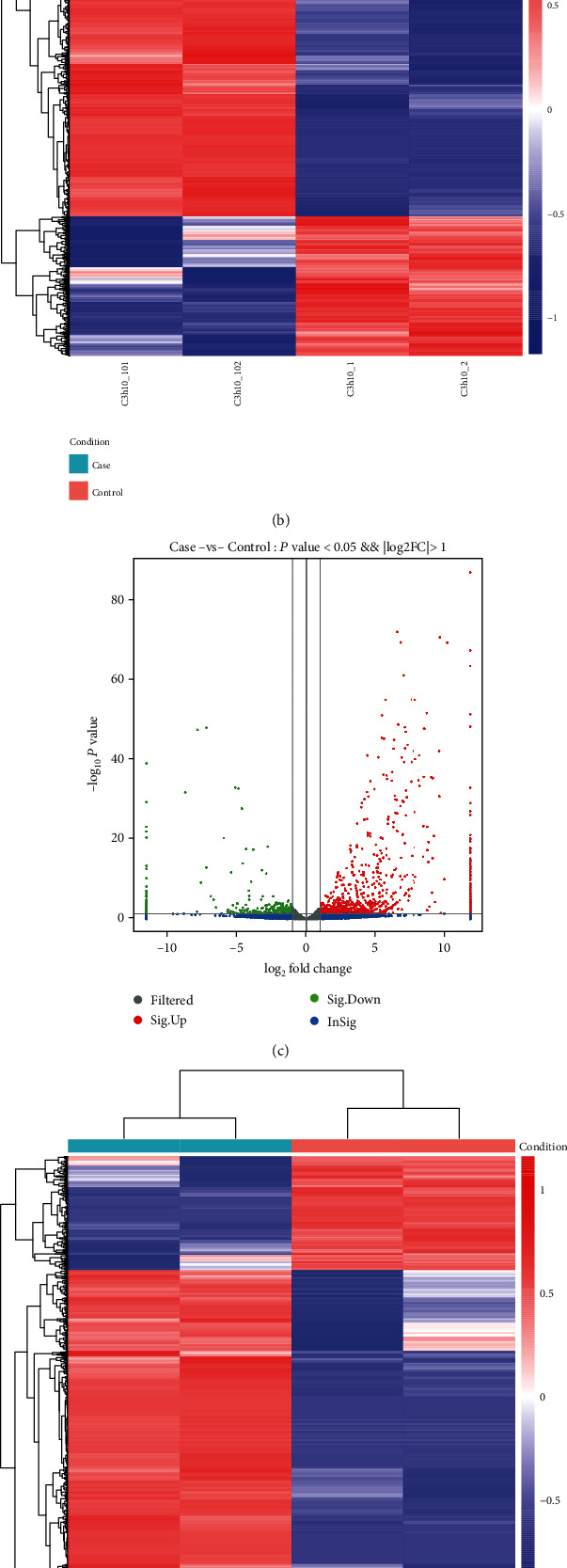
Differential expression profiles of mRNA-lncRNAs between undifferentiated and differentiated MSCs. (a) The volcano figure of the differentially expressed mRNAs between 5-AZA-treated and untreated cells, respectively (C3h10_1 and C3h10_2: control; C3h10_101 and C3h10_102: case). (b) Differential gene expression profiling was identified by hierarchical clustering analysis, indicating that undifferentiated and differentiated stem cells exhibited dramatically differentially expressed gene profile (C3h10_1 and C3h10_2: control; C3h10_101 and C3h10_102: case). (c) The volcano figure of the differentially expressed lncRNAs between 5-AZA-treated and untreated cells, respectively (C3h10_1 and C3h10_2: control; C3h10_101 and C3h10_102: case). (d) Differential lncRNA profiling was identified by hierarchical clustering analysis, indicating that undifferentiated and differentiated stem cells exhibited a dramatically lncRNA expression profile (C3h10_1 and C3h10_2: control; C3h10_101 and C3h10_102: case). (e) Statistics of lncRNA types showed that there are (i) 240 intergenic lncRNAs (u), (ii) 197 intronic lncRNAs (i), (iii) 105 anti-sense lncRNAs (x), (iv) 365 sense-overlapping lncRNA intergenic (o).

By calculating the RFPKM values and comparing the sequences, we screened 3398 differentially expressed mRNAs, including 2429 upregulated and 969 downregulated mRNAs, according to the criteria of false discovery rate (FDR) < 0.05 and ∣logFC | >1 (Figure 5(a)). Meanwhile, differential gene expression profiling was identified by hierarchical clustering analysis, confirming that undifferentiated and differentiated stem cells exhibited dramatically differentially expressed gene profile (Figure 5(b)).

Using the same sequencing profiling with lncRNAs, we found 1538 differentially expressed lncRNAs, suggesting different kinds of lncRNAs expression were caused by myogenic and cardiogenic differentiation (Figure 5(c)). In addition, 1175 and 363 lncRNAs were identified significantly up- and downregulated, respectively (FDR ≤ 0.05, fold change ≥ 2) (Figure 5(d)). The differential lncRNAs found in this study were classified into: (i) sense, (ii) antisense, (iii) bidirectional, (iv) intronic, and (v) intergenic (Figure 5(e)).

### 3.3. Functional Annotation and Pathway Analysis of Differential mRNA

**Figure 6:**
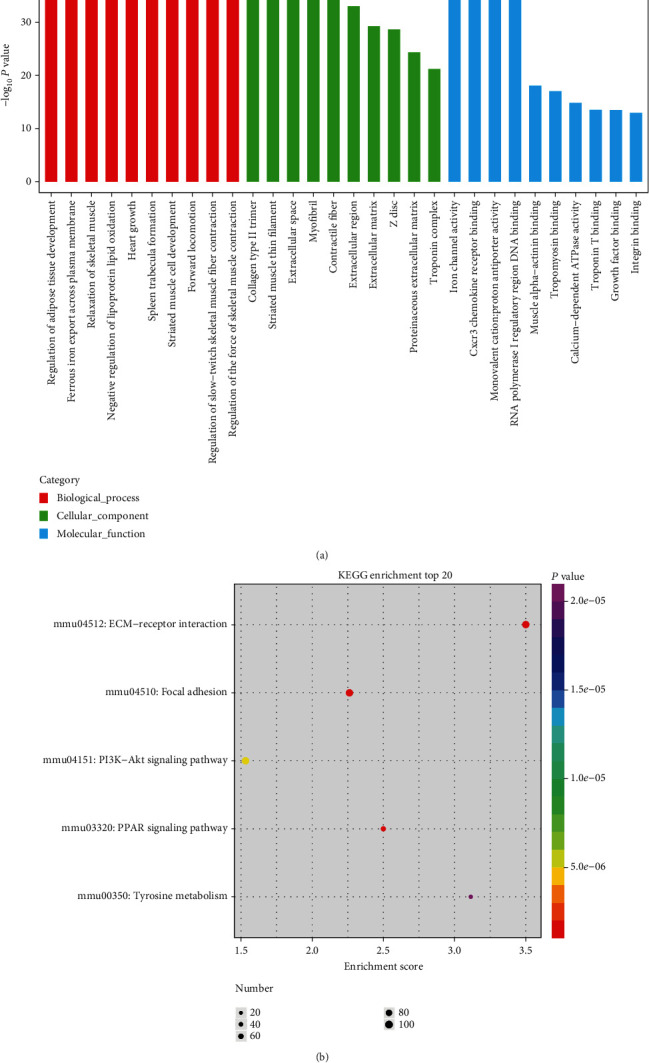
Differential mRNA functional annotation and pathway analysis between undifferentiated and differentiated MSCs. (a) GO analysis. (b) KEGG analysis.

To understand the related biological processes, we performed Genetic Ontology (GO) and Kyoto Encyclopedia of Genes and Genomics (KEGG) pathway analysis. GO and KEGG enrichment analysis showed that there are significantly more genes are related to cardiac muscle differentiation (*P* ≤ 0.00). GO analysis revealed abundantly expressed mRNAs involved in upregulated GO function, including heart growth, striated muscle cell development, regulation of the force of skeletal muscle contraction, striated muscle thin filament, Z disc, troponin complex, and muscle alpha-actinin binding and *ect* (Figure 6(a)). Pathway analysis showed that mRNA expression was involved in significantly upregulated pathways, including those for focal adhesion, ECM-receptor interaction, PI3K-AKT signaling pathway, PPAR signaling pathway, and tyrosine metabolism (Figure 6(b)).

### 3.4. Identification of Differentially Expressed circRNAs on Myogenic and Cardiomyogenic Differentiation of MSCs and Analysis of circRNA-Targeted Differentially Expressed Genes

**Figure 7:**
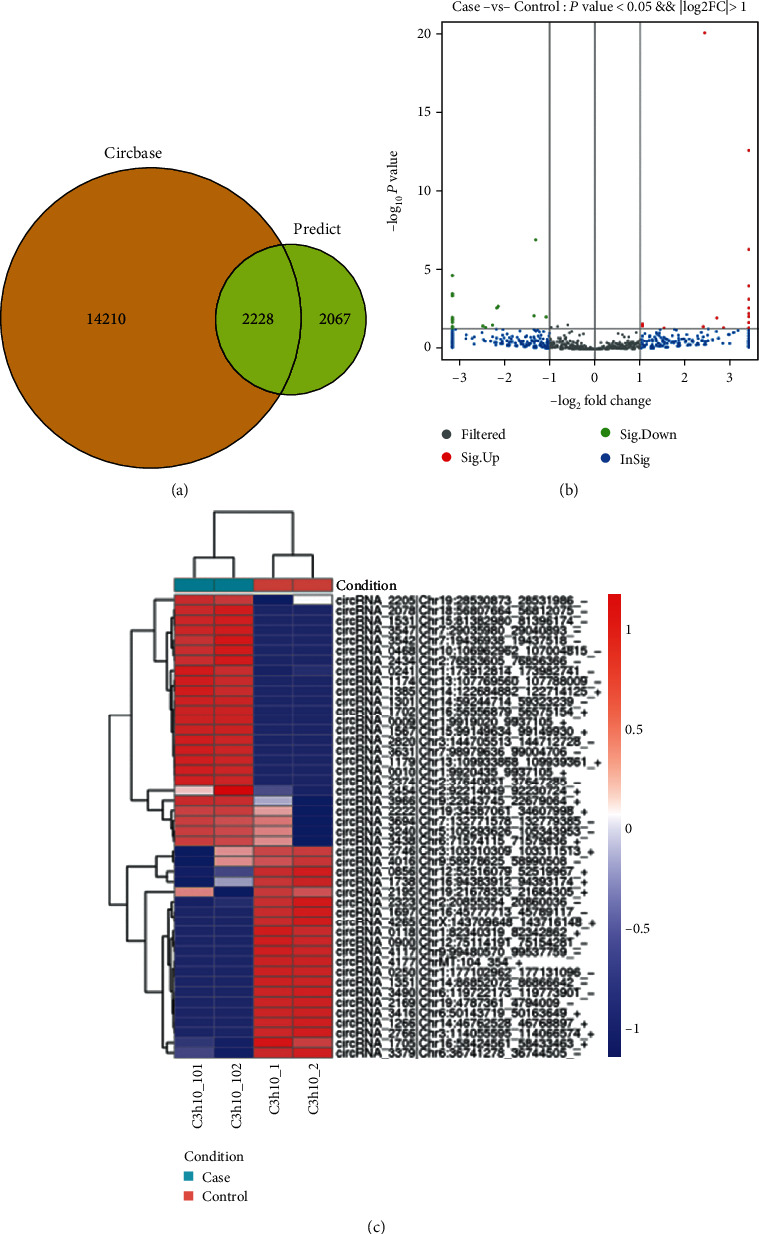
Differential expression of circRNAs between undifferentiated and differentiated MSCs (C3h10_1 and C3h10_2: control; C3h10_101 and C3h10_102: case). (a) Venn diagram showing the number of expression patterns of circRNAs. (b) Volcano plots of circRNAs. Volcano plots were constructed by fold-change and *P* values. The vertical lines correspond to *P* values, and the horizontal lines represent log2 (fold change) up- and downregulation between control versus case samples. The red (up) and green (down) points show the significantly differentially expressed circRNAs. (c) Hierarchical clustering of circRNAs. Hierarchical clustering analysis of circRNAs that was differentially expressed between control versus case samples (fold change > 2; ^∗^*P* < 0.05).

High-throughput sequencing technology was used to detect the expression profile of circRNAs, and a total of 2228 circRNAs were identified (Figure 7(a)) when compared with the known database circBase (http://www.circbase.org/). In addition, we found that a total of 46 circRNAs were differentially expressed, among which 25 and 21 lncRNAs were identified significantly up- and downregulated, respectively (**FDR** ≤ 0.05, **fold** **change** ≥ 2) (Figure 7(b)). Moreover, differential circRNA expression profiling was identified by hierarchical clustering analysis, confirming that undifferentiated and differentiated stem cells exhibited dramatically differentially expressed circRNAs profile (Figure 7(c)).

**Figure 8:**
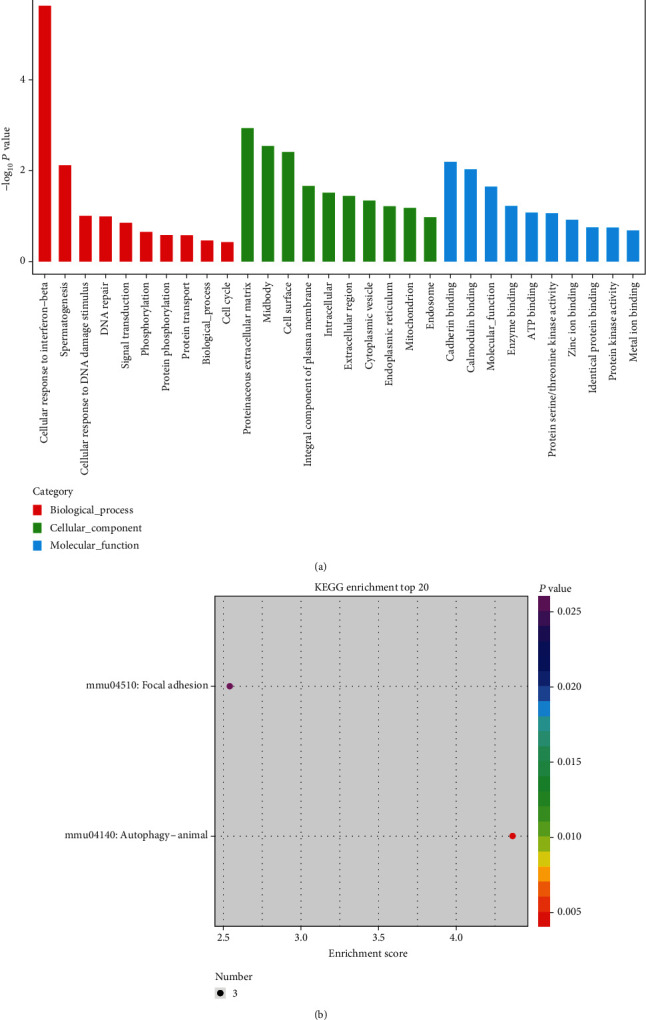
Differential cirRNA functional annotation and pathway analysis between undifferentiated and differentiated MSCs. (a) GO analysis. (b) KEGG analysis.

To analy**ze** and predict the putative function of differentially expressed circRNAs on myogenic differentiation of MSCs, GO categories and KEGG pathway analyses were performed on the circRNA-host genes (Figures 8(a) and 8(b)). We analyzed the upregulated target mRNAs of circRNA-targeted differentially expressed genes to determine their differences in molecular function. The target mRNAs were found to play vital roles in biological regulation, catalytic activity, developmental process, and metabolic process including cellular response to interferon-beta, proteinaceous extracellular matrix, and cadherin binding. In addition, upregulated KEGG pathways of circRNA-targeted differentially expressed genes were found to participate mainly in **f**ocal adhesion and **a**utophagy.

### 3.5. GSEA Analysis

**Figure 9:**
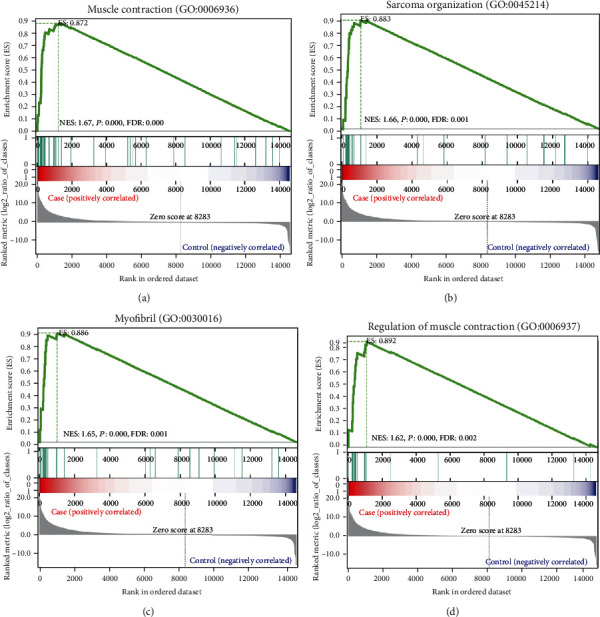
Four GO enrichment graphs of GSEA results. (a) Muscle contraction (*P* = 0.00, FDR = 0.00); (b) sarcomere organization (*P* = 0.00, FDR = 0.0007); (c) myofibril (*P* = 0.00, FDR = 0.0005); (d) regulation of muscle contraction (*P* = 0.00, FDR = 0.0023).

The GO pathway involved in the whole gene expression level of GSEA was further explored and verified. As shown in the table, 20 significantly enriched pathways were identified (FDR < 0.25, Table 3) including muscle contraction (GO: 0006936), myofibril (GO: 0030016), sarcomere organization (GO: 0045214), and regulation of muscle contraction (GO: 0006937) (*P* < 0.01, FDR < 0.25). Moreover, muscle contraction (GO: 0006936), sarcomere organization (GO: 0045214), myofibril (GO: 0030016), and regulation of muscle contraction (GO: 0006937) were displayed in Figure 9.

### 3.6. The Selected lncRNAs and circRNAs Were Verified by qRT-PCR

**Figure 10:**
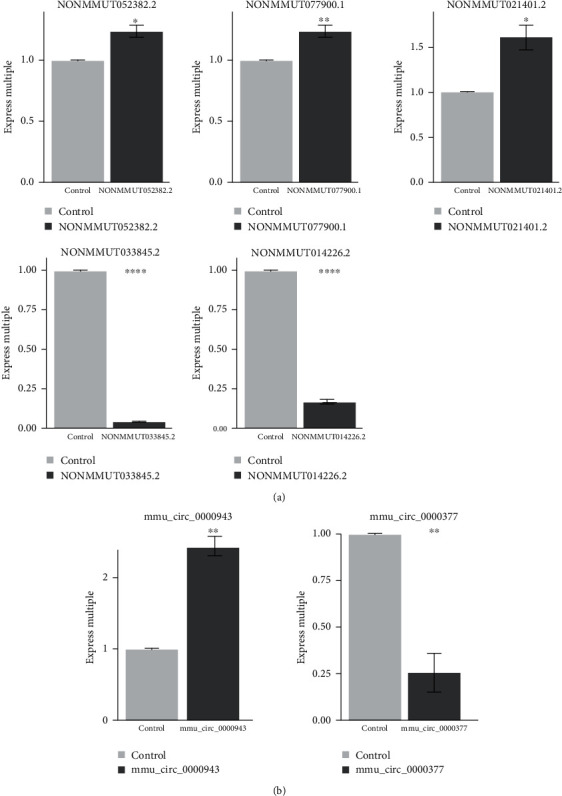
qRT-PCR validation results of selected lncRNAs and circRNAs. (a) Expression levels of the 5 selected lncRNAs verified by qPCR. (b) The expression levels of the 2 selected circRNAs verified by qPCR.

In order to verify the RNA sequencing results of lncRNAs and circRNAs, we selected 8 lncRNAs and 3 circRNAs totally. Our data indicate that 5 lncRNAs were consistent with the results of the RNA-seq experiment, including 3 upregulated lncRNAs: NONMMU052382.2 (*P* < 0.01), NONMMU077900.1 (*P* < 0.01), and NONMMU021401.2 (*P* < 0.01) and 2 downregulated lncRNAs: NONMMU033845.2 (*P* < 0.01) and NONMMU014226.2 (*P* < 0.01) (Figure 10(a)). In addition, 2 of 3 circRNAs were consistent with the results of the RNA-seq experiment, of which mmu_circ_0000943 (*P* < 0.01) was upregulated, and mmu_circ_0000377 (*P* < 0.01) was downregulated (^∗^*P* < 0.05, ^∗∗^*P* < 0.01; Figure 10(b)).

## 4. Discussion

The potential of C3H10T1/2 cells to differentiate into myocytes and cardiomyocytes using the small molecules 5-AZA that epigenetically regulate epigenetic gene expression patterns was evaluated in the present study. Morphologically, after 5-AZA treatment, fibroblast-like cells gradually enlarged to a myotubular-like structure. To enhance the efficiency, we used a method “cell ring” to pick up the cell clone of myotube-like cell masses. However, this method is more likely a very effective way to improve cell purity than cell quantity. We found that these myotube-like cells still have abilities to form myotube-like structure, indicating that these cells have relatively stable differentiation status and limited proliferation abilities. No beating cardiomyocytes were observed in all of our experimental groups. In addition, 5-AZA was found to be an effective chemical reagent to induce the differentiation of stem cells, as was proven by a change in the myogenic and cardiac-specific marker expression. The expression level of cardiac-specific and myogenic-specific markers including *GATA4*, *cTnT*, *MyoD*, and *Desmin* is significantly upregulated which is consistent with the data of the previous research. ICC results showed that myogenic and cardiogenic markers, *Desmin*, *GATA4*, *MyoD1*, and *TNNT2*, were significantly upregulated in the differentiated cells induced by 5-AZA than the untreated group of MSCs, suggesting that 5-AZA could promote the myogenic and cardiogenic differentiation of MSCs. Our data suggest that a stable in vitro myogenic and cardiac differentiation model can be established by 5-AZA. Moreover, we noticed that the expression level of *GATA4* increased more than 100 times (^∗∗^*P* < 0.01) after 5-AZA treatment. *GATA4* has been shown to control the differentiation and growth of many kinds of cells [18, 19]. In the process of myocardial cell differentiation, the quantity of GATA4 transfected bone marrow MSCs was higher than that of control cells, suggesting that GATA4 increased the differentiation potential of stem cells into myocardial cells. It has been shown that MSCs could be induced into cardiomyocyte-like cells by TGF-*β*1 or 5-AZA [20]. The low dose treatment of both TGF-*β*1 and 5-AZA could shorten the induction time to 2 weeks compared to that by using only 5-AZA. TGF-*β*1 induced a lower differentiation efficiency than 5-AZA in MSCs, while 5-AZA exhibited apparent toxicity phenomenon during cell induction processes. The detailed mechanisms underlying the myogenic and cardiac differentiation of MSCs need to be further explored.

Stem cell therapy is a potential way for heart disease [21, 22], one of the crucial causes of morbidity and mortality in the world [23]. However, insufficient quantity and quality of cardiomyocytes derived from stem cells have become one of the major problems to achieve an effective treatment. Research has shown that the differentiation of stem cells into cardiomyocytes is fundamentally dependent on complex cellular and molecular mechanisms [24, 25]. The classical view of gene regulation focuses on protein-coding genes. Recently, ncRNAs, including lncRNAs and circRNAs, have been identified for their potential roles as central regulators of cell-specific gene networks [26]. lncRNAs are widely defined as regulated noncoding transcripts over 200 nucleotides in length and have become important regulators of cellular function through a variety of functional roles. Recent findings suggest that multiple lncRNAs have been identified as regulators of cardiac pedigree specification and differentiation [27–29]. It mediates heart commitment epigenetics and plays important roles in the determination of stem cells into the heart [30]. However, a complete analysis of the expression of lncRNAs in the control of cardiac differentiation and function in stem cells has not been fully reported until now.

Basically, lncRNAs are expressed in a manner of highly dependent on cell type or tissue specificity ways [31], and they are less conserved at the sequence level than protein-coding RNAs [32]. The expression patterns of many lncRNAs show multiform pathophysiologic appearances in different tissue and condition. We therefore employed an integrated genomic approach to identify the differentially expressed lncRNAs, and circRNAs in 5-AZA induced cardiac and myogenic differentiation of a well-characterized cell line C3H10T1/2, which enabled us to identify ncRNAs of interest that may lay the foundation for further studies on myogenic and cardiac differentiation.

Through high-throughput techniques and bioinformatics analysis, we identified differentially expressed, known, and novel lncRNAs that may play a role in myogenic and cardiac differentiation. First, we have identified 2429 and 969 mRNAs up- and downregulated in undifferentiated and differentiated MSCs in this study. We further verified the experimental results of RNA-seq by qRT-PCR. The qRT-PCR results showed that among the 8 randomly selected lncRNAs and 3 circRNAs, 5 lncRNAs and 2 circRNAs were in line with the experimental results of RNA-seq, among which 3 lncRNAs were upregulated (NONMMU052382.2, NONMMU077900.1, and NONMMU021401.2), 2 were downregulated (NONMMU033845.2 and NONMMU014226.2), 1 circRNA was upregulated (mmu_circ_0000943), and 1 circRNA was downregulated (mmu_circ_0000377). The experimental results of the other 3 lncRNAs (NONMMUT026407.2, NONMMUT033845.2, and NONMMUT003617.2) and 1 circRNAs (mmu_circ_0001047) were not significant or did not conform to the experimental results of RNA-seq, which requires further experimental verification.

Enrichment analysis of GO and KEGG showed that the significantly upregulated genes are related to cardiac and muscle differentiation, metabolic process, developmental process, and catalytic activity. This suggested that different kinds of gene expression were caused by cardiogenic and myogenic differentiation. GO analysis showed that many gene expressions were involved in the upregulation of GO function, including heart growth, striated muscle cell development, regulation of the force of skeletal muscle contraction, striated muscle thin filament, Z disc, troponin complex, and muscle alpha-actinin binding. Furthermore, we also assume that focal adhesion, ECM-receptor interaction, PI3K-AKT signaling pathway, PPAR signaling pathway, and tyrosine metabolism might be involved in the processes.

The GSEA results further confirmed the identified pathways (such as muscle contraction (GO: 0006936), sarcomere organization (GO: 0045214), myofibril (GO: 0030016), and regulation of muscle contraction (GO: 0006937)). GSEA analysis showed that C3H10T1/2 showed obvious characteristics of myoblast differentiation after induction, and the results showed that the pathway was indeed related to muscle contraction. Those novel biological processes identified by GSEA help us better understand the molecular mechanisms by which lncRNAs regulate the presence of MSCs. Finally, on the basis of GO enrichment analysis, KEGG path analysis, and GSEA results, we found that there were obvious similarities between the different analysis results.

Meanwhile, differentially expressed lncRNAs in undifferentiated and differentiated stem cells were evaluated by RNA sequencing profiling. Moreover, 1175 and 363 lncRNAs were identified significantly up- and downregulated, respectively. There are more known than novel lncRNAs among the differentially expressed lncRNAs, indicating that hundreds of lncRNAs were involved in the process of cardiac and muscle differentiation. These lncRNAs are widely distributed on all chromosomes, among which sense-overlapping and intergenomic lncRNAs account for the majority of lncRNAs. In addition, many lncRNAs are shorter, have lower expression levels, and are less conserved than the protein-coding transcripts [33]. Our results showed that many lncRNAs were significantly correlated with large amounts of mRNAs. Taking together, 1538 differentially expressed lncRNAs were found in this study during 5-AZA-stimulated differentiation for 21 days in C3H10T1/2 cells. Although more study is needed to prove the exact role and mechanism of lncRNAs in cardiomyogenic differentiation, lncRNAs seem to be an effective candidate for future myogenic and cardiomyogenic differentiation or heart diseases therapy.

In recent years, little research has been undertaken on the expression profiles of circRNAs in myogenic and cardiomyogenic differentiation of stem cells. A total of 46 differentially expressed circRNAs were identified, including 25 upregulated and 21 downregulated circRNAs in the present study. The subsequent functional analysis of these circRNAs will be helpful to understand the myogenic and cardiac differentiation of stem cells. Recently, it has been proposed that circRNAs can accommodate miRNA and found that it is rich in functional miRNA-binding sites [34]. Furthermore, GO and pathway analysis showed that these target mRNAs play an important role in biological regulation, catalytic activity, developmental processes, and metabolic processes, including cellular response to interferon-*β*, proteinaceous extracellular matrix, and cadherin combined response. In addition, the upregulation of the KEGG pathway targeting circRNA differentially expressed genes is mainly involved in focal adhesion and autophagy.

In summary, we have described the expression profiles of lncRNAs and circRNAs that may affect the myogenic and cardiomyogenic differentiation of mouse mesenchymal stem cells. Our findings may provide a theoretical basis for future studies on ncRNA regulation of myogenic and cardiac differentiation. This result provides a basis for further experimental study on the function and mechanism of lncRNAs and circRNAs in stem cell differentiation. It is necessary to conduct follow-up investigations to learn more information about the role of ncRNA in myogenic and cardiac differentiation. We speculate that differentially expressed ncRNAs, including lncRNAs and circRNAs, can represent new molecular targets for clinical treatment of muscle-related diseases.

## 5. Conclusions

To sum up, our study found significantly differential expression of ncRNAs in myogenic and myogenic differentiation of MSCs. The present study provided a systematic perspective on the expression of ncRNAs during myogenic and cardiomyogenic differentiation of MSCs. This work provides convincing evidence that the identified lncRNAs and circRNAs are potential biomarkers for stem cells to undergo myogenic and cardiac differentiation. However, further studies on the target verification and functional analysis of these lncRNAs and circRNAs can help provide conclusive evidence to explain the regulatory mechanism of lncRNAs and circRNAs in the differentiation process of mouse MSCs.

## Figures and Tables

**Table 1:** Primers used in this study.

Gene	Primer (5′ →3′)
GATA-4	Forward: TCT CAC TAT GGG CAC AGC AG Reverse: CGA GCA GGA ATT TGA AGA GG

CTNT	Forward: CAG AGG AGG CCA ACG TAG AAG Reverse: CTC CAT CGG GGA TCT TGG GT

*α*-MHC	Forward: CCA CTG TGG TGC CTC GTT C Reverse: GCG TCC GTC ATT CTG TCA CTC

MYOD	Forward: GGG TTC CCT GTT CTG TGT CGC TT Reverse: CGC TCC AAC TGC TCT GAT GGC AT

MYOGENIN	Forward: AGT GAA TGC AAC TCC CAC AG Reverse: ACG ATG GAC GTA AGG GAG TG

DESMIN	Forward: GTG AAG ATG GCC TTG GAT GT Reverse: GTA GCC TCG CTG ACA ACC TC

GAPDH	Forward: ACC ACA GTC CAT GCC ATC AC Reverse: TCC ACC ACC CTG TTG CTG TA

**Table 2:** Primers used in this study.

Gene	Primer (5′ →3′)	Up_down
GAPDH_mouse	Forward: TGATGGGTGTGAACCACGAG Reverse: GGTCATGAGCCCTTCCACAA	

NONMMUT013708.2	Forward: TGTCCTGAGCCATGGGTAGA Reverse: GGAGCAGCTATGAGCACAGT	Up

NONMMUT052382.2	Forward: ATAACCCTACCCCTAGCCCC Reverse: TGGGATGAAGTCCTACAGTCCT	Up

NONMMUT026407.2	Forward: ATGGGTGGTTATGCGTGTGT Reverse: CATAGCGTTCTCGTCCACCA	Up

NONMMUT077900.1	Forward: CTGCAATGGAAAGGCTCTGC Reverse: TTTCCCAGAGCAACCCTGAC	Up

NONMMUT021401.2	Forward: GCTCTCTTGCTTCTCGCTCT Reverse: GCCAGGAAGAACACCACAGA	Up

NONMMUT033845.2	Forward: TGAAGGTTGGACCCGTGAAG Reverse: TCTCACCTCGAGCACCAAAC	Down

NONMMUT014226.2	Forward: GTGGCCGGCTTGTATGACAT Reverse: TGCATTTCACAACGCCTGTT	Down

NONMMUT003617.2	Forward: TGTCCTTGTCACCATCCTGC Reverse: TGCCTTCCACACGCTATTGT	Down

circRNA_0856 (mmu_circ_0000377)	Forward: TGCAATCACATCTGACCAGGA Reverse: CCCTCATTGCCAAAGAAAGGTC	Down

circRNA_2205 (mmu_circ_0000943)	Forward: AGAGTCTCTGGTGTCCACGA Reverse: GGGAACTGTGGCTGGATGAA	Up

circRNA_2454 (mmu_circ_0001047)	Forward: TGGTTGCCCAAATGAAGCAG Reverse: GCTCCTTCAGCTCTCCAGTC	Up

**Table 3:** The significant enriched GO pathways from GSEA results (^∗∗^*P* < 0.01, FDR < 0.25).

Term_ranked	Term	ES	NES	*P* value	FDR	Geneset_size	Matched_size
1	Muscle contraction (GO: 0006936)	0.872	1.669	0	0	51	46
2	Sarcomere organization (GO: 0045214)	0.883	1.658	0	0.001	39	33
3	Myofibril (GO: 0030016)	0.886	1.654	0	0.001	41	31
4	Oogenesis (GO: 0048477)	0.879	1.632	0	0.002	38	27
5	Regulation of muscle contraction (GO: 0006937)	0.892	1.623	0	0.002	22	20
6	Z disc (GO: 0030018)	0.79	1.594	0	0.007	122	98
7	Condensed nuclear chromosome (GO: 0000794)	0.827	1.583	0	0.012	43	38
8	Striated muscle thin filament (GO: 0005865)	0.904	1.577	0	0.014	21	16
9	Myosin complex (GO: 0016459)	0.818	1.573	0	0.015	47	37
10	A band (GO:0031672)	0.879	1.564	0	0.022	22	18
11	M band (GO: 0031430)	0.882	1.562	0.001	0.022	24	17
12	Synaptonemal complex (GO: 0000795)	0.859	1.541	0	0.045	29	22
13	Structural constituent of cytoskeleton (GO: 0005200)	0.779	1.535	0	0.05	71	59
14	I band (GO: 0031674)	0.869	1.535	0	0.052	23	20
15	Striated muscle contraction (GO: 0006941)	0.881	1.526	0.001	0.064	23	16
16	Cardiac muscle tissue morphogenesis (GO: 0055008)	0.863	1.51	0.006	0.103	18	16
17	Structural constituent of muscle (GO: 0008307)	0.845	1.507	0.001	0.109	28	22
18	Microtubule-based process (GO: 0007017)	0.798	1.505	0.004	0.107	35	31
19	Skeletal muscle fiber development (GO: 0048741)	0.831	1.503	0.002	0.106	27	22
20	Cardiac muscle contraction (GO: 0060048)	0.797	1.501	0	0.111	50	40

## Data Availability

The data set generated and/or analyzed in the current research process is included in the article and can be obtained from the corresponding author under reasonable request. The RNA-Seq analysis data have been deposited in the GEO public database repository (accession number GSE168981).
